# Depression of synaptic outputs by reduction of Ca^2+^ influx, but not RRP vesicles, becomes evident upon CB2R hyper-expression at cerebellar Purkinje cell terminals

**DOI:** 10.3389/fnmol.2026.1842304

**Published:** 2026-08-13

**Authors:** Takuma Inoshita, Shin-ya Kawaguchi

**Affiliations:** 1 Department of Biophysics, Graduate School of Science, Kyoto University, Kyoto, Japan; 2 Department of Physiology, Kitasato University School of Medicine, Kanagawa, Japan

**Keywords:** cannabinoid, CB2R, GPR55, Purkinje cell, synaptic suppression, transmitter release

## Abstract

Synaptic transmission is dynamically regulated by neuromodulators. One well-studied example is cannabinoid receptor type 1 (CB1R)-mediated suppression of neurotransmitter release by reducing presynaptic Ca^2+^ influx. Recently, GPR55 was shown to regulate transmitter release through a mechanism distinct from that of CB1R. However, the presynaptic role of cannabinoid receptor type 2 (CB2R) remains unclear. Here we studied this issue using cerebellar Purkinje cells (PCs), which are known to express both CB2R and GPR55 and amenable to direct patch-clamp recordings from axon terminals. At naïve PC synapses onto target neurons, application of a CB2R agonist did not affect synaptic transmission. The lack of effect was ascribed to minimal endogenous CB2R at distal axon terminals of PCs. Exogenous expression of CB2R enabled suppression of synaptic transmission upon pharmacological activation. Direct voltage-clamp recordings of presynaptic Ca^2+^ current and membrane capacitance changes at boutons demonstrated that CB2R activation reduced presynaptic Ca^2+^ influx without affecting the total amount of readily releasable vesicles, leading to less vesicle exocytosis through lowered release probability. Notably, replacement of two intracellular loops of CB2R with those of GPR55 converted the site of action for synaptic suppression from Ca^2+^ channel inhibition to the reduction of the readily releasable vesicles, as GPR55 does. These findings indicate that, when sufficiently present at axon terminals, CB2R suppresses transmitter release through reduction of Ca^2+^ influx like CB1R, and suggest that intracellular loops of cannabinoid receptors determine whether transmitter release is suppressed through inhibition of presynaptic Ca^2+^ channels or reduction of the readily releasable pool of vesicles.

## Introduction

Synaptic transmission is dynamically regulated by neuromodulators, among which cannabinoid signaling has been extensively studied. Activation of cannabinoid receptor type 1 (CB1R) suppresses transmitter release by inhibiting presynaptic Ca^2+^ influx (Kreitzer and Regehr, 2001; Ohno-Shosaku et al., 2001; Wilson and Nicoll, 2001; Diana et al., 2002), which lowers release probability (Pr) within the classical quantal hypothesis proposed by del Castillo and Katz (1954). The molecular basis of CB1R-mediated synaptic suppression was established by Kano and colleagues through a series of studies on retrograde endocannabinoid signaling (Ohno-Shosaku et al., 2002; Hashimotodani et al., 2005; Hashimotodani et al., 2007). More recently, another cannabinoid-related receptor, GPR55, has also been shown to regulate transmitter release through a distinct mechanism. We demonstrated that, at cerebellar Purkinje cell (PC) axon terminals, GPR55 suppresses transmitter release by reducing the size of the readily releasable pool (RRP) of synaptic vesicles, corresponding to the number of release-ready vesicles (N), without altering presynaptic Ca^2+^ influx (Inoshita and Kawaguchi, 2026). However, because these mechanisms of synaptic modulation (Pr vs. N regulation) have only been studied in different types of neurons, it remains unclear whether distinct cannabinoid receptors can regulate transmitter release through separate presynaptic mechanisms within a single presynaptic terminal.

To examine this question, PCs are suitable because they express multiple cannabinoid receptors, including GPR55 and cannabinoid receptor type 2 (CB2R), whereas CB1R is absent (Gong et al., 2006; Cabral and Griffin-Thomas, 2009; Stella, 2010; Sánchez-Zavaleta et al., 2018; Inoshita and Kawaguchi, 2026). Although CB2R was originally identified in immune cells and microglia (Munro et al., 1993; Cabral and Griffin-Thomas, 2009; Atwood and Mackie, 2010), it is now also recognized to be expressed in neurons, where accumulating evidence indicates roles in neuronal activity (Zhang et al., 2014; Li and Kim, 2016). In PCs, postsynaptic CB2Rs regulate inhibitory synaptic transmission (Sadanandan et al., 2020). Nevertheless, whether and how CB2R regulates presynaptic transmitter release remains largely unknown. Although CB2R mRNA has been detected in PCs, conventional cannabinoid receptor (CB1R/CB2R)-mediated suppression of transmitter release is absent at PC axon terminals (Hirono and Yanagawa, 2020) whereas GPR55-dependent suppression is clearly present (Inoshita and Kawaguchi, 2026). We therefore asked whether the lack of conventional cannabinoid-mediated modulation reflects insufficient presynaptic localization of CB2Rs, or specialization of PC axon for GPR55-mediated presynaptic modulation. Because PCs allow genetic manipulation together with direct presynaptic patch-clamp recordings from axonal boutons (Kawaguchi and Sakaba, 2015), we could address this question using the gain-of-function strategy previously applied in cultured hippocampal neurons (Atwood et al., 2012).

Here, we demonstrated that the exogenously expressed CB2R suppresses transmitter release by inhibiting presynaptic Ca^2+^ influx without affecting the RRP when present at presynaptic boutons, indicating that the lack of cannabinoid-mediated modulation primarily reflects insufficient localization of endogenous CB2R. Furthermore, replacing intracellular loops of CB2R with those of GPR55 converted the mechanism of presynaptic suppression from Ca^2+^ channel inhibition (Pr) to RRP reduction (N). These results indicate that distinct cannabinoid receptors can regulate transmitter release through different presynaptic mechanisms within the same neuronal terminal.

## Materials and methods

### Animal usage

All experimental procedures were performed in accordance with regulations on animal experimentation at Kyoto University and approved by the local committee in the Graduate School of Science, Kyoto University. Wistar rats (Slc: Wistar; Japan Slc Inc.) of either sex were used in this study. Rats were killed by decapitation.

### Slice preparation

Acute sagittal slices (200 μm thickness) of the cerebellum were prepared from Wistar rats at P14-21. Rats were anesthetized with isoflurane and perfused transcardially with ice-cold sucrose solution containing (in mM): NaCl 60, sucrose 120, NaHCO_3_ 25, NaH_2_PO_4_ 1.25, KCl 2.5, D-glucose 25, ascorbic acid 0.4, myo-inositol 3, Na-pyruvate 2, CaCl_2_ 0.1, MgCl_2_ 3, pH 7.3–7.4, 300–350 mOsm/kg H_2_O continuously bubbled with mixed gas (95% O_2_ and 5% CO_2_). The cerebellum was removed and cut with a Leica vibroslicer (VT1200S) in an ice-cold sucrose solution. Slices were then incubated at 37 °C for a 0.5–1 h in an extracellular solution containing (in mM): NaCl 125, NaHCO_3_ 25, NaH_2_PO_4_ 1.25, KCl 2.5, D-glucose 25, ascorbic acid 0.4, myo-inositol 3, Na-pyruvate 2, CaCl_2_ 2, MgCl_2_ 1, pH 7.3–7.4, 300–320 mOsm/kg H_2_O continuously bubbled with mixed gas.

### Cerebellar primary cultures

Primary dissociated cultures of cerebellar neurons were prepared as described previously (Kawaguchi and Hirano, 2007). Briefly, cerebella were dissected out from newborn rats and their meninges were removed. The cerebella were incubated in Ca^2+^ and Mg^2+^-free Hank’s balanced salt solution containing 0.1% trypsin and 0.05% DNase for 15 min at 37 °C. Cells were dissociated by trituration and seeded in Dulbecco’s modified Eagle’s medium: nutrient mixture F12-based medium containing 2% fetal bovine serum. Medium was replaced with Basal Medium Eagle on the following day and half-changed every 3–4 days. Cytosine arabinoside (4 mM) was added to the medium to inhibit proliferation of glial cells. PCs were identified by large cell bodies and thick dendrites. Experiments were performed > 21 days after preparation of the culture.

### DNA construction and transfection

Plasmids encoding EGFP were identical to those used in previous studies (Kawaguchi and Sakaba, 2015; Inoshita and Kawaguchi, 2026). The coding sequence of CB2R (RefSeq NM_001164142.4) or GPR55 (RefSeq XM_006245494.4) fused to Venus at the C-terminal was inserted into the pAAV-CA-WPRE plasmid (Higashi et al., 2024). PCs were transduced with CB2R- or GPR55-Venus using adeno-associated virus serotype 2 (AAV2) at 1–7 days after seeding. A chimera receptor was generated by PCR-based DNA modification to replace two intracellular regions of CB2R (415–438 and 679–690 bases) by the corresponding sequences of GPR55 (379–399 and 631–642 bases). Plasmids encoding the chimera were directly injected into the nucleus of PCs through sharp glass pipettes (Kawaguchi and Hirano, 2007) together with an EGFP plasmid to identify transfected neurons. Experiments were carried out 1–2 days after the injection.

### Electrophysiology

Patch-clamp recordings were performed using EPC10 amplifier (HEKA) at room temperature (20–24 °C), in an extracellular solution described above for slices, or that containing the following for culture (in mM): NaCl 145, HEPES 10, glucose 10, CaCl_2_ 2, MgCl_2_ 1, pH 7.3–7.4 adjusted with KOH, and 300–310 mOsm/kg H_2_O. In some experiments, 2,3-Dioxo-6-nitro-1,2,3,4-tetrahydrobenzo[f]quinoxaline-7-sulfonamide (NBQX, 10 μM), tetrodotoxin (TTX, 1 μM) and tetraethylammonium (TEA, 2 mM) were applied to the bath. Internal solutions contained (in mM): current-clamp, KCl 147, HEPES 10, EGTA 0.5, Mg-ATP 2, Na-GTP 0.2; postsynaptic voltage-clamp, CsCl 147, HEPES 10, EGTA 0.5, Mg-ATP 2, Na-GTP 0.2; bouton voltage-clamp, CsCl 103, KCl 44, HEPES 10, EGTA 0.5, Mg-ATP 2, Na-GTP 0.2. All solutions were adjusted to pH 7.3–7.4 with KOH (current-clamp) or CsOH (voltage-clamp) and 320–330 mOsm/kg H_2_O. PCs were held at −70 mV unless otherwise stated. PCs’ target postsynaptic neurons were voltage clamped at −70 to −100 mV in slices, or at −70 mV in culture for inhibitory postsynaptic currents (IPSC) recordings. On-line series resistance compensation (20–60%) was applied for presynaptic terminal recordings. In paired whole-cell recordings from a presynaptic PC soma and its postsynaptic neuron, action potentials (APs) were elicited by voltage pulses to 0 mV for 1–5 ms into PC soma. In the slice, IPSCs were evoked by a glass pipette electrode placed in the white matter. The amplitude and frequency of miniature IPSCs (mIPSCs) were calculated from > 200 events (> 7 pA). Membrane capacitance (C_m_) was measured using sine + DC technique (Neher and Marty, 1982) implemented in PatchMaster (HEKA). Presynaptic terminals were held at −80 mV and the sine wave (1 kHz and the peak amplitude of 30 mV) was superimposed on the holding potential. Because membrane conductance fluctuates during the depolarizing pulse, C_m_ was usually measured approximately 50 ms after the depolarization. Bouton size was estimated from capacitive transients evoked by 5–10 mV hyperpolarizing pulses. To calibrate the variation in presynaptic Ca^2+^ current (I_Ca2+_) and neurotransmitter release depending on the size of the bouton, I_Ca2+_ and ΔC_m_ were normalized by the C_m_ under the voltage-clamp in each bouton. Images were obtained with a Quantalux sCMOS camera (Thorlabs) on IX71 or BX51WI microscopes (Olympus).

### Immunocytochemistry

Cultured neurons were fixed with 4% paraformaldehyde in phosphate-buffered saline, permeabilized with 0.5% Tween-20, blocked with 2% skim milk, and labeled with primary and secondary antibodies. The following antibodies were used: mouse monoclonal antibody (Ab) against calbindin (1:1000; Swant, #300REC) and rabbit Ab against CB2R (1:1000; Alomone Labs, ACR-002), Alexa Fluor 568-conjugated Ab against rabbit immunoglobulin G (IgG) (1:1000, Thermo Fisher Scientific, A-11011), and Alexa Fluor 488-conjugated Ab against mouse IgG (1:1000; Thermo Fisher Scientific, A-11039). Fluorescent images were recorded using a confocal laser microscope (FV1000 imaging system, Olympus) and analyzed using ImageJ software (NIH).

### Statistics

Data are presented as mean ± SEM. Statistical analyses used paired or unpaired Student’s *t*-test, Dunnett’s test or two-way ANOVA. *p* < 0.05 was considered significant.

## Results

### The absence of cannabinoid-mediated suppression reflects insufficient presynaptic localization of CB2R

**Figure 1:**
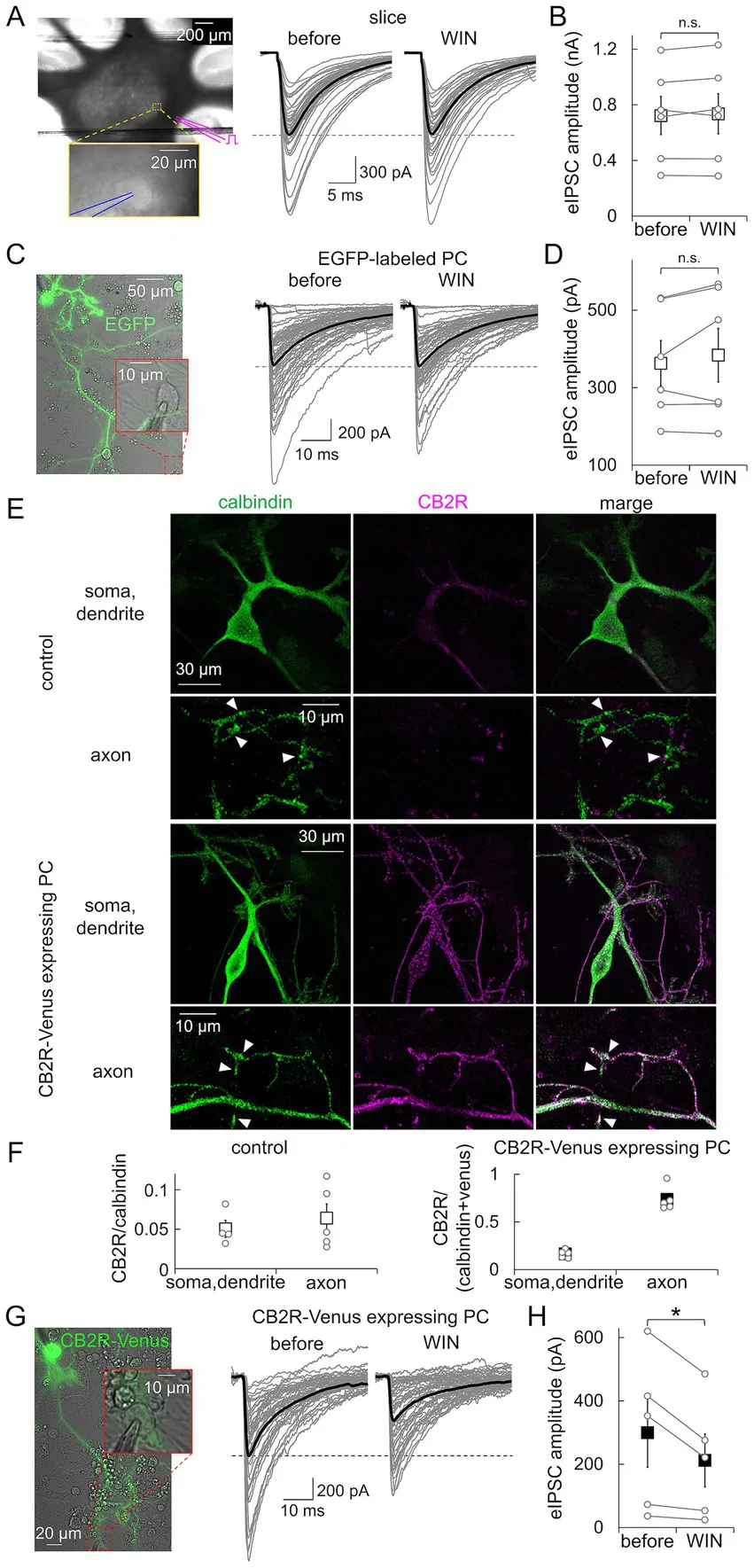
Exogenous expression of CB2R suppresses transmitter release. **(A)** Image of patch-clamp recording from a DCN neuron in a slice (left). A magnified image of the recorded neuron is shown as an inset (a patch pipette is indicated in blue). A pipette for electrical stimulation (magenta) was placed at the white matter. Individual (gray) and averaged (black) traces (right) of eIPSC before and 5 min after the application of WIN. **(B)** eIPSC amplitude before and after the WIN55,212–2 (WIN, 300 nM) application, *n* = 6. **(C)** Image of dual whole-cell recordings from a presynaptic EGFP-labeled PC soma and its postsynaptic target neuron in culture (left). Representative traces of eIPSCs before and 5 min after the application of WIN (right). **(D)** eIPSC amplitude before and after the WIN application, *n* = 6. **(E)** Images of immunostained CB2R (magenta) and calbindin (a marker of PC, green) in control (top) and CB2R-Venus expressing PCs (bottom). White arrowheads indicate axonal varicosities of PCs. **(F)** Fluorescence intensity of CB2R signal relative to calbindin or to the sum of calbindin and Venus measured in the somatodendritic and axonal regions. Control: somatodendritic region, *n* = 4 cells; axonal region, *n* = 5. CB2R-Venus expressing PC: somatodendritic region, *n* = 5; axonal region, *n* = 5. **(G)** Image of dual whole-cell recordings from a presynaptic CB2R-Venus expressing PC soma and its postsynaptic target neuron in culture (left). Representative traces of eIPSCs before and 5 min after the application of WIN (right). **(H)** eIPSC amplitude before and after the WIN application, *n* = 5. Data are mean ± *SEM*. *p < 0.05; n.s., not significant.

To determine why conventional cannabinoid receptor-mediated suppression is absent at PC output synapses, we first re-examined the effect of cannabinoid receptor agonist WIN55,212–2 (WIN) on PC to deep cerebellar nuclei (DCN) transmission in acute cerebellar slices prepared from Wistar rats at P14-21. Whole-cell patch-clamp recordings were obtained from large DCN neurons (> 15 μm in diameter) voltage-clamped at −70 to −100 mV (Uusisaari et al., 2007; Hirono et al., 2018) (Figure 1A). Evoked IPSCs (eIPSCs) were elicited by electrical stimulation of the cerebellar white matter in the presence of NBQX (10 μM). eIPSCs at PC-DCN synapses exhibited large amplitudes (0.72 ± 0.14 nA) and an almost all-or-none response, consistent with previous descriptions of PC-DCN synapses (Kawaguchi and Sakaba, 2015). To determine whether CB2R (or CB1R, if any) modulates transmission, we applied the CB1R/CB2R agonist WIN (300 nM), which did not significantly change eIPSC amplitude (0.74 ± 0.14 nA, 5 min after the application of WIN, *p* = 0.423, paired *t*-test) (Figures 1A,B). These results are consistent with previous reports that PC-DCN synapses are insensitive to WIN in acute slices (Hirono and Yanagawa, 2020). We next examined the effect of WIN in dissociated cerebellar cultures, which permit subsequent genetic manipulation (Kawaguchi and Sakaba, 2015). PCs were labeled using AAV2-CA-EGFP, which preferentially infects PCs in this preparation (Kawaguchi and Sakaba, 2015). Paired recordings were obtained from an EGFP-positive presynaptic PC and a postsynaptic neuron surrounded by EGFP-labeled boutons in the presence of NBQX (10 μM) (Figure 1C). Presynaptic PCs were voltage-clamped at −70 mV, and APs were elicited by brief voltage pulse (to 0 mV, 1 ~ 5 ms), evoking IPSCs in the voltage-clamped postsynaptic neuron. As in slice recordings, WIN (300 nM) did not significantly affect IPSC amplitude (363 ± 59 to 384 ± 69 pA, before or 5 min after the application of WIN, *p* = 0.293, paired *t*-test) (Figures 1C,D), confirming that cannabinoid receptor-mediated suppression is absent at naïve PC-DCN synapses both in slices and in culture.

Although CB2R mRNA has been detected in PCs and CB2R-positive fibers have been reported in the DCN (Gong et al., 2006; Cabral and Griffin-Thomas, 2009; Stella, 2010; Sánchez-Zavaleta et al., 2018), direct evidence for CB2R localization at PC axon terminals is lacking. We therefore examined the subcellular distribution of CB2R in cerebellar cultures by immunocytochemistry. CB2R immunoreactivity was observed in PC soma and dendrites, together with weak labeling near the axon initial segment (Figure 1E). In contrast, CB2R immunoreactivity was barely detectable at distal axonal boutons (Figure 1E). We next exogenously expressed CB2R in PCs to promote its localization to axons (Atwood et al., 2012) and examine whether CB2R suppresses synaptic transmission when localized to presynaptic boutons. CB2R-Venus was exogenously expressed in PCs using an AAV vector, and punctate CB2R-Venus signals were observed not only in soma and dendrites but also along axons and at axon terminals (Figures 1E,F). Paired recordings from CB2R-Venus expressing PCs and their postsynaptic target neurons showed that eIPSCs amplitudes were comparable to control cells (300 ± 109 pA, *p* = 0.701, compared with EGFP-labeled PCs, Student’s *t*-test). In contrast, application of WIN significantly suppressed synaptic transmission from CB2R-Venus expressing PCs (212 ± 83 pA, 5 min after the application of WIN, *p* = 0.0407, paired *t*-test) (Figures 1G,H). This suppressive effect of WIN was abolished by the CB2R antagonist AM630 (3 μM) in CB2R-Venus expressing PCs (328 ± 58 pA to 358 ± 72 pA, *p* = 0.498, paired *t*-test) (Supplementary Figure S1), indicating that the observed synaptic suppression by WIN was mediated by the exogenously expressed CB2R. These results suggest that the lack of CB2R-mediated synaptic modulation at naïve PC-DCN synapses is explained primarily by the absence of endogenous CB2R at presynaptic terminals and indicate that CB2R can mediate presynaptic inhibition when present at PC axon terminals.

### Reduction of presynaptic Ca^2+^ influx underlies CB2R-mediated synaptic suppression

**Figure 2:**
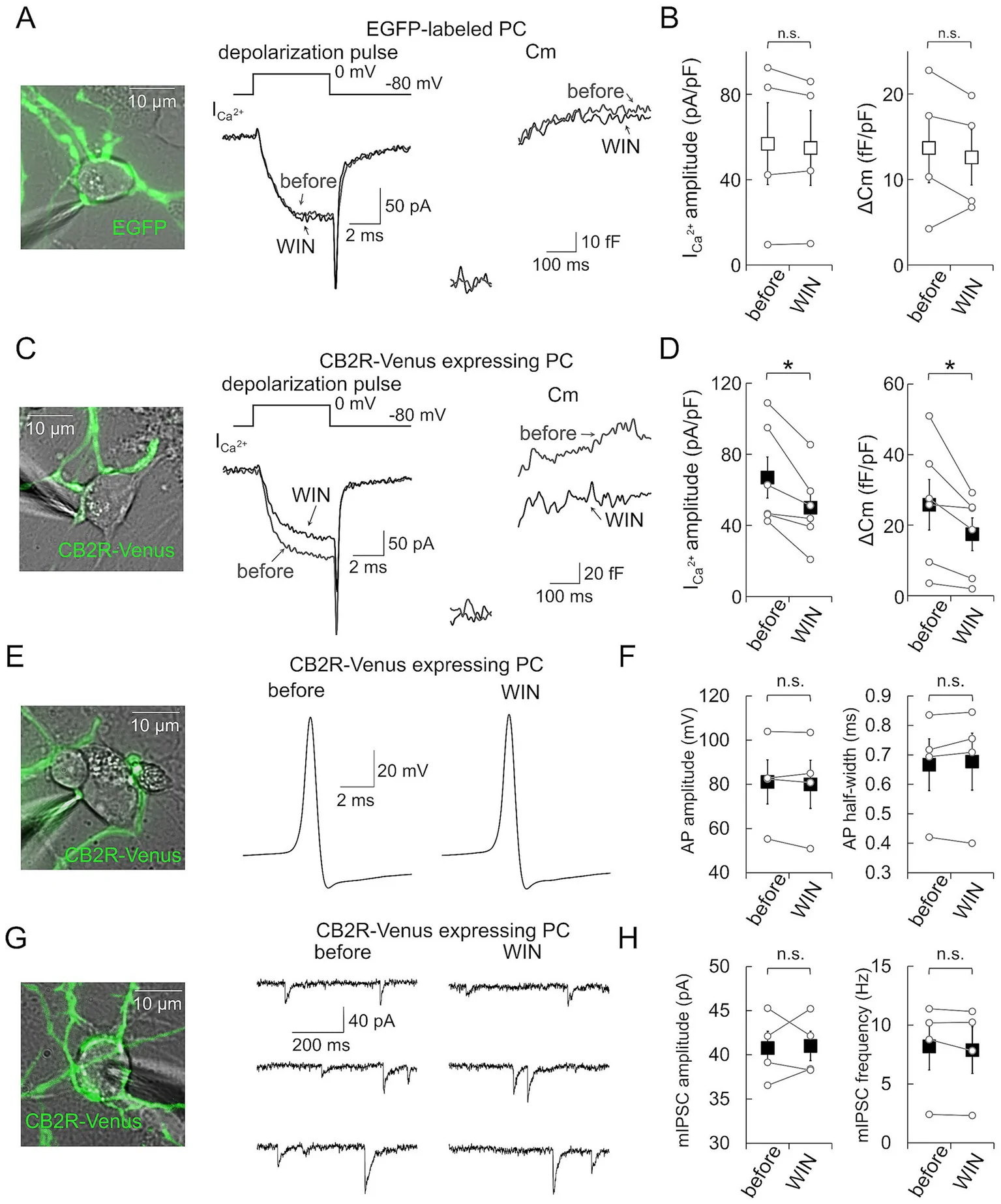
Exogenous CB2R enables suppression of vesicle exocytosis via reduced Ca^2+^ influx. **(A)** Image of direct patch-clamp recording from an EGFP-labeled PC axon terminal in culture (left). Representative traces of presynaptic Ca^2+^ currents (I_Ca_^2+^) during 5 ms depolarization and the resultant C_m_ increase (ΔC_m_) before and after the WIN application (right). **(B)** Amplitude of I_Ca_^2+^ and ΔC_m_ before and after the WIN application in EGFP-labeled PCs, *n* = 4. **(C)** Image of direct patch-clamp recording from a CB2R-Venus expressing PC axon terminal in culture (left). Representative traces of I_Ca_^2+^ and ΔC_m_ before and after the WIN application (right). **(D)** Amplitude of I_Ca_^2+^ and ΔC_m_ before and after the WIN application in CB2R-Venus expressing PCs, *n* = 6. Both I_Ca_^2+^ and ΔC_m_ were normalized by the size of presynaptic C_m_ under the voltage-clamp. **(E,F)** Image of recording from axon terminal of CB2R-Venus expressing PCs and representative traces **(E)**, amplitude and half-width **(F)** of APs before and after the WIN application, *n* = 4. **(G,H)** Image of mIPSCs recording from target cell of CB2R-Venus expressing PCs and representative traces **(G)**, amplitude and frequency **(H)** of mIPSCs before and after the WIN application, *n* = 4. Data are mean ± SEM. *p < 0.05; n.s., not significant.

To investigate the mechanism underlying CB2R-mediated synaptic suppression, we next examined presynaptic Ca^2+^ influx because CB2R is a G_i/o_-coupled receptor like CB1R (Atwood and Mackie, 2010). For this purpose, we performed patch-clamp recordings from individual PC boutons to simultaneously measure I_Ca2+_ and vesicle exocytosis. I_Ca2+_ was evoked by a 5 ms depolarizing pulse to 0 mV in the presence of TTX (1 mM) and TEA (2 mM), and exocytosis of vesicles was monitored simultaneously as changes in C_m_ (ΔC_m_), which reflects the increase in plasma membrane area following synaptic vesicle fusion. In control PC boutons expressing EGFP with an AAV vector, depolarizing pulses induced I_Ca2+_ (57 ± 19 pA/pF) accompanied by increases in C_m_ (14 ± 4 fF/pF). Application of WIN did not affect either the I_Ca2+_ amplitude or ΔC_m_ (I_Ca2+_: 55 ± 18 pA/pF, *p* = 0.380, paired *t*-test; ΔC_m_: 13 ± 3 fF/pF, *p* = 0.451, paired *t*-test) (Figures 2A,B). Similarly, the CB2R-selective agonist CB65 (30 nM) did not significantly affect either presynaptic I_Ca2+_ current or ΔC_m_ in naïve PCs (I_Ca2+_: 44 ± 16 to 42 ± 16 pA/pF, *p* = 0.125, paired *t*-test; ΔC_m_: 21 ± 9 to 21 ± 7 fF/pF, *p* = 0.873, paired *t*-test) (Supplementary Figure S2). In contrast, PC boutons expressing CB2R-Venus showed marked reduction in I_Ca2+_ after the application of WIN (67 ± 12 to 50 ± 9 pA/pF, *p* = 0.0193, paired *t*-test), which was accompanied by a decrease in ΔC_m_ (26 ± 7 to 17 ± 5 fF/pF, *p* = 0.0482, paired *t*-test) (Figures 2C,D). These results indicate that CB2R suppresses transmitter release through inhibition of presynaptic Ca^2+^ influx, thereby reducing Pr.

To determine whether mechanisms other than reduced presynaptic Ca^2+^ influx contribute to the CB2R-mediated synaptic suppression, we next examined AP propagation and postsynaptic responsiveness. CB2R activation affected neither AP waveform (amplitude: 81.1 ± 10.0 to 80.0 ± 11.0 mV, *p* = 0.483, paired *t*-test; half-width, 0.67 ± 0.09 to 0.68 ± 0.10 ms, *p* = 0.397, paired *t*-test) (Figures 2E,F) nor mIPSCs (amplitude: 41 ± 2 to 41 ± 2 pA, *p* = 0.874, paired *t*-test; frequency, 8.2 ± 2.0 to 7.9 ± 2.0 Hz, *p* = 0.264, paired *t*-test) (Figures 2G,H), indicating that reduced presynaptic Ca^2+^ influx is the primary mechanism underlying CB2R-mediated suppression of transmitter release.

### RRP size is insensitive to CB2R activation

**Figure 3:**
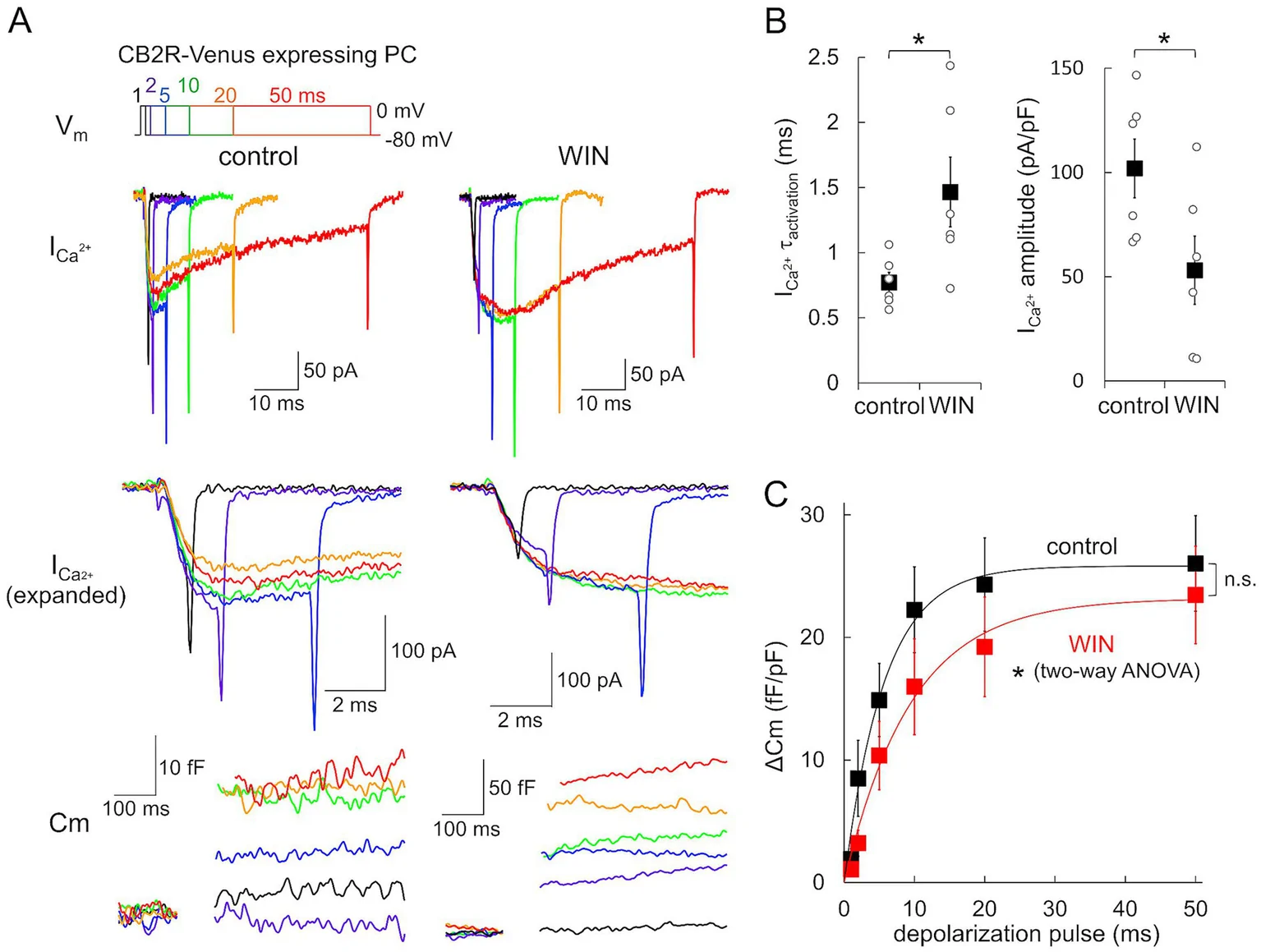
CB2R activation suppresses presynaptic vesicle exocytosis without affecting the RRP size. **(A)** Representative traces of *I*_Ca2+_, expanded *I*_Ca2+_, and the resultant Δ*C*_m_ upon 1, 2, 5, 10, 20, or 50 ms of depolarization pulses (to 0 mV) without (left) or with (right) WIN. **(B)** Time constant for activation and amplitude of *I*_Ca2+_ without (*n* = 6) or with WIN (*n* = 6). **(C)** Δ*C*_m_ upon depolarization pulses recorded without (black, *n* = 8) or with WIN (red, *n* = 8). Single exponential fits for each are shown as lines. Both *I*_Ca2+_ and *C*_m_ change were normalized by the size of presynaptic *C*_m_ under the voltage-clamp. Data are mean ± SEM. *p < 0.05; n.s., not significant.

Because CB2R suppresses transmitter release by reducing Pr through inhibition of presynaptic Ca^2+^ influx, we next examined whether CB2R also regulates another key parameter, N, corresponding to the RRP size, as observed for GPR55 (Inoshita and Kawaguchi, 2026). To estimate the RRP size, we recorded I_Ca2+_ and ΔC_m_ while systematically varying the duration of depolarizing pulses. Increasing the duration of depolarization prolonged I_Ca2+_ and produced corresponding increases in ΔC_m_ (Figure 3A). In CB2R-Venus expressing boutons, ΔC_m_ tended to mono-exponentially increase with increasing pulse duration, reaching a plateau during prolonged depolarizations (20–50 ms), corresponding to depletion of RRP. The relationship between pulse duration and ΔC_m_, as well as the estimated RRP size, was comparable to previous reports from PC axon terminals (Kawaguchi and Sakaba, 2015; Inoshita and Kawaguchi, 2026). WIN slowed I_Ca2+_ activation (τ_activation_) (0.77 ± 0.08 to 1.47 ± 0.27 ms; *p* = 0.0481, Student’s *t*-test) (Figure 3B), consistent with previous reports of G_i/o_-mediated modulation of voltage-gated Ca^2+^ channels (VGCCs) (Dolphin, 2003; Zamponi and Currie, 2013). In addition, the I_Ca2+_ peak evoked by prolonged depolarizations was smaller in the presence of WIN (101.9 ± 14.1 to 53.1 ± 16.4 pA/pF; *p* = 0.0471, Student’s *t*-test) (Figure 3B). Despite the marked reduction in presynaptic I_Ca2+_, ΔC_m_ evoked by a 50-ms depolarization with or without WIN at axonal boutons of CB2R-Venus expressing PCs was not significantly different (26 ± 4 vs. 23 ± 4 fF/pF; *p* = 0.651, Student’s *t*-test) (Figure 3C), indicating that CB2R activation does not alter the RRP. It should be noted that the ΔC_m_ evoked by shorter depolarizing pulses was smaller with WIN (*p* = 0.0261, two-way ANOVA), in accord with the slowed activation and reduced amplitude of presynaptic I_Ca2+_. Together, these results indicate that CB2R suppresses transmitter release through inhibition of presynaptic Ca^2+^ influx without altering the RRP, indicating selective regulation of Pr rather than N, unlike GPR55.

### Replacement of CB2R intracellular loops with those of GPR55 converts its action from lowering Pr to N

**Figure 4:**
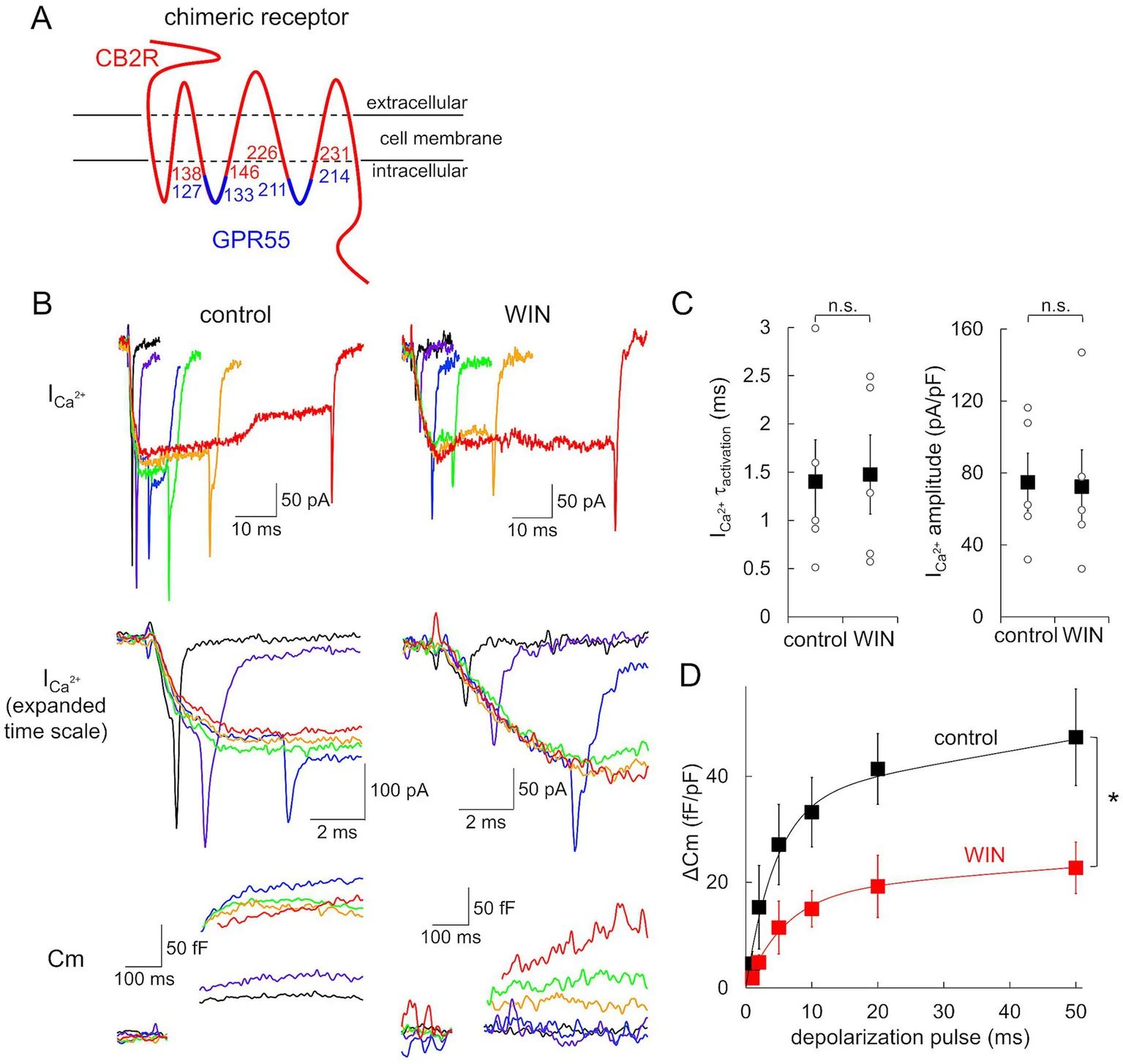
Activation of chimeric receptor with WIN reduces the size of RRP. **(A)** Schematic illustration of the chimera in which the second and third intracellular regions of CB2R (red) were replaced with the corresponding sequences of GPR55 (blue). Numbers indicate the corresponding amino acid positions in CB2R and GPR55. **(B)** Representative traces of I_Ca2+_, expanded I_Ca2+_, and the resultant ΔC_m_ upon 1, 2, 5, 10, 20, or 50 ms of depolarization pulses (to 0 mV) without (left) or with (right) WIN. **(C)** Time constant for activation and amplitude of I_Ca2+_ without (*n* = 5) or with WIN (*n* = 5). **(D)** ΔC_m_ upon depolarization pulses recorded without (black, *n* = 7) or with WIN (red, *n* = 6). Data were fitted with the sum of an exponential and a linear component (lines). Both I_Ca2+_ and C_m_ change were normalized by the size of presynaptic C_m_ under the voltage-clamp. Data are mean ± SEM. *p < 0.05; n.s., not significant.

The above results showed that CB2R and GPR55 suppress transmitter release through distinct presynaptic mechanisms. We next asked whether this difference is determined by receptor-specific intracellular signaling. Because CB2R and GPR55 are thought to be coupled to different G-protein subtypes (Lauckner et al., 2008; Atwood and Mackie, 2010), we generated a chimeric receptor by replacing the second and third intracellular loops of CB2R, which contribute to G-protein coupling specificity (Weis and Kobilka, 2018; Zhang et al., 2020), with the corresponding regions of GPR55 (Figure 4A). The chimera was expressed in PCs by microinjection of plasmid DNA into the nucleus, together with an EGFP plasmid to identify transfected neurons. Direct patch-clamp recordings were then performed from chimera-expressing PC axon terminals. Activation kinetics and peak amplitude of the presynaptic I_Ca2+_ were not affected by WIN (τ_activation_: 1.40 ± 0.43 to 1.48 ± 0.41 ms, *p* = 0.905; I_Ca2+_ amplitude: 74.8 ± 16.1 to 72.4 ± 20.4 pA/pF, *p* = 0.929, Student’s *t*-test) (Figure 4C), indicating that replacement of two intracellular loops abolished CB2R-mediated inhibition of presynaptic I_Ca2+_. We next analyzed the relationship between pulse duration and ΔC_m_ (Figures 4B,D). In terminals expressing the chimera, ΔC_m_ evoked by a 50-ms depolarization with WIN was smaller than that without WIN (Figure 4D) (47 ± 9 vs. 23 ± 5 fF/pF; *p* = 0.0440, Student’s *t*-test). The ΔC_m_-pulse duration relationships were fitted by the sum of single exponentials and linear components (control, y = 36(1 − e^-x/4^) + 0.22x; WIN, y = 18(1 − e^-x/6^) + 0.10x, respectively) (Figure 4D). The linear components are likely to reflect the slowly releasable pool or the rate of vesicle replenishment (Sakaba and Neher, 2001a; Lee et al., 2010). The fast component of RRP, estimated from exponential fit, was reduced approximately by half in the presence of WIN. This effect resembled the RRP reduction previously observed following GPR55 activation (Inoshita and Kawaguchi, 2026). Together, these results suggest that the cannabinoid receptor subtype, through its intracellular loops, determines whether transmitter release is suppressed through inhibition of presynaptic Ca^2+^ influx (Pr) or reduction of the RRP (N).

## Discussion

The present study addressed two related questions regarding cannabinoid signaling at PC axon terminals. First, the absence of conventional cannabinoid receptor-mediated suppression at naïve PC boutons primarily reflects insufficient presynaptic localization of endogenous CB2R. Second, when localized to presynaptic boutons, CB2R suppresses transmitter release by inhibiting presynaptic Ca^2+^ influx (Pr) without altering the readily releasable pool (N), in contrast to GPR55.

### Presynaptic localization limits conventional cannabinoid signaling at PC boutons

Our results indicate that presynaptic localization, rather than receptor function itself, limits conventional cannabinoid signaling at naïve PC axon terminals. The physiological functions of G protein-coupled receptors (GPCRs) are critically influenced by their subcellular distribution. For example, the C-terminal intracellular region of CB1R regulates axonal targeting and surface expression, thereby influencing receptor function (Fletcher-Jones et al., 2019). Although CB2R possesses a structurally similar C-terminal region (Haspula and Clark, 2020), whether it contributes to axonal trafficking or presynaptic localization remains unknown. Because endogenous CB2R was barely detectable at distal PC axon terminals, its subcellular localization may be tightly regulated. In the present study, exogenously expressed CB2R-Venus was detected not only in somatodendritic compartments but also in axons. Because Venus was fused to the C terminus, a region implicated in regulating the subcellular distribution of CB1R (Fletcher-Jones et al., 2019), this manipulation may have affected presynaptic localization of CB2R. Alternatively, increased CB2R expression may be sufficient to promote presynaptic localization, because overexpression of wild-type CB2R resulted in its localization to presynaptic terminals in cultured hippocampal neurons (Atwood et al., 2012). These possibilities highlight the importance of clarifying the molecular mechanisms governing CB2R subcellular localization for understanding how physiological cannabinoid signaling is regulated at presynaptic terminals.

### Distinct cannabinoid receptors selectively regulate different quantal parameters of transmitter release

Our direct presynaptic recordings indicate that CB2R suppresses transmitter release through the canonical G_i/o_-dependent mechanism of presynaptic Ca^2+^ channel inhibition, similar to CB1R, GABA_B_ receptors, and group II/III metabotropic glutamate receptors (Takahashi et al., 1998; Kew et al., 2001; Bowery et al., 2002; Hirono and Yanagawa, 2020). In contrast, GPR55 regulates transmitter release by reducing the RRP, without affecting presynaptic Ca^2+^ influx (Inoshita and Kawaguchi, 2026). Together, these findings demonstrate that CB1R/CB2R-like and GPR55-like mechanisms can operate within the same presynaptic terminal.

Our chimera experiments suggest that intracellular loops of cannabinoid receptors contribute to determining whether presynaptic suppression is mediated through regulation of Pr or N. Because GPR55 has been proposed to couple to G_q_ and G_12/13_ proteins (Lauckner et al., 2008; Atwood and Mackie, 2010), the observed switch in presynaptic inhibitory mechanism might reflect coupling to a different G_α_ subtype. However, the present study did not directly identify the G-protein subtype coupled to the chimera. Future studies combining direct analyses of G-protein coupling with identification of the downstream signaling pathways will be required to determine how intracellular loops of cannabinoid receptors regulate distinct presynaptic mechanisms. An additional technical limitation of the present study is that the subcellular localization of the chimera was not directly examined. Nevertheless, because both CB2R-Venus and GPR55-Venus were detected in axons following exogenous expression (Figures 1E,G and Supplementary Figure S3), the chimera was also likely to reach presynaptic boutons.

Although both mechanisms ultimately suppress transmitter release, regulation of Pr and N is expected to have different functional consequences. Modulation of Pr primarily influences the initial efficacy and short-term plasticity of synaptic transmission, whereas regulation of the RRP is expected to influence the ability of synapses to sustain transmitter release during repetitive activity (Sakaba and Neher, 2001b; Zucker and Regehr, 2002; Regehr, 2012; Neher, 2024). Thus, CB1R/CB2R-like and GPR55-like signaling may differentially influence neural circuit function depending on the temporal pattern of synaptic activity.

### Potential physiological and pathological consequences of presynaptic CB2R recruitment

Under basal conditions, GPR55 appears to be the predominant cannabinoid receptor regulating transmitter release at PC boutons because conventional cannabinoid receptor-mediated modulation is absent (Hirono and Yanagawa, 2020; Inoshita and Kawaguchi, 2026). However, this situation may not be static. CB2R expression is markedly increased during neuroinflammation, neuronal injury, and several neurological disorders (Cabral and Griffin-Thomas, 2009; Zhang et al., 2014). If this increase is accompanied by recruitment of CB2R to presynaptic boutons, cannabinoid signaling could regulate both Pr and N within the same synapse, potentially expanding the dynamic range of presynaptic modulation during prolonged neuronal activity or pathological conditions.

Recruitment of presynaptic CB2R may also alter activity-dependent cannabinoid signaling. At PC–DCN synapses, DSI has not been observed (Hirono and Yanagawa, 2020), and GPR55 is suggested not to be activated by classical endocannabinoids such as 2-arachidonoylglycerol or anandamide (Oka et al., 2007). However, if CB2R is recruited to axon terminals, conventional endocannabinoid-dependent plasticity, including DSI, could emerge at PC-DCN synapses. Future studies examining whether endogenous CB2R is recruited to PC boutons under pathological conditions, and how it interacts with GPR55, will help clarify state-dependent regulation of cerebellar output.

## Data Availability

The original contributions presented in the study are included in the article/Supplementary material, further inquiries can be directed to the corresponding author.

## Supplementary material

SUPPLEMENTARY FIGURE S1 AM630 abolished WIN-induced synaptic suppression in CB2R-Venus expressing PC. **(A)** Image of dual whole-cell recordings from a presynaptic CB2R-Venus expressing PC soma and its postsynaptic target neuron in culture (left). Representative traces of eIPSCs before and 5 minutes after the application of WIN in the presence of AM630 (right). **(B)** eIPSC amplitude before and after the WIN application in the presence of AM630, *n* = 4. Data are mean ± SEM. n.s., not significant.

SUPPLEMENTARY FIGURE S2 CB65, a CB2R specific agonist, induced no detectable change on Ca^2+^ influx and transmitter release. **(A)** Representative traces of presynaptic I_Ca2+_ during 5 ms depolarization and the resultant ΔC_m_ before and after the CB65 application (right). **(B)** Amplitude of I_Ca2+_ and ΔC_m_ before and after the CB65 application in EGFP-labeled PCs. *n* = 4. Data are mean ± SEM. n.s., not significant.

SUPPLEMENTARY FIGURE S3 Exogenously expressed GPR55-Venus is present in PC axons. Representative images of PCs expressing exogenous GPR55-Venus. A patch pipette is indicated in blue.
